# A cationic lipid mediated CRISPR/Cas9 technique for the production of stable genome edited citrus plants

**DOI:** 10.1186/s13007-022-00870-6

**Published:** 2022-03-18

**Authors:** Lamiaa M. Mahmoud, Prabhjot Kaur, Daniel Stanton, Jude W. Grosser, Manjul Dutt

**Affiliations:** 1 Citrus Research and Education Center, University of Florida, Lake Alfred, FL 33850 USA; 2 Pomology Department, Faculty of Agriculture, Mansoura University, Mansoura, Egypt; 3 Department of Horticulture, Michigan State University, East Lansing, MI 48824 USA

**Keywords:** Citrus, Protoplast, CRISPR/Cas9, Genome editing, Lipofection, Systemic acquired resistance (SAR), *NPR3*

## Abstract

**Background:**

The genetic engineering of crops has enhanced productivity in the face of climate change and a growing global population by conferring desirable genetic traits, including the enhancement of biotic and abiotic stress tolerance, to improve agriculture. The clustered regularly interspaced short palindromic repeats (CRISPR/Cas9) system has been found to be a promising technology for genomic editing. Protoplasts are often utilized for the development of genetically modified plants through in vitro integration of a recombinant DNA fragment into the plant genome. We targeted the citrus Nonexpressor of Pathogenesis-Related 3 (*CsNPR3*) gene, a negative regulator of systemic acquired resistance (SAR) that governs the proteasome-mediated degradation of NPR1 and developed a genome editing technique targeting citrus protoplast DNA to produce stable genome-edited citrus plants.

**Results:**

Here, we determined the best cationic lipid nanoparticles to deliver donor DNA and described a protocol using Lipofectamine™ LTX Reagent with PLUS Reagent to mediate DNA delivery into citrus protoplasts. A Cas9 construct containing a gRNA targeting the *CsNPR3* gene was transfected into citrus protoplasts using the cationic lipid transfection agent Lipofectamine with or without polyethylene glycol (PEG, MW 6000). The optimal transfection efficiency for the encapsulation was 30% in Lipofectamine, 51% in Lipofectamine with PEG, and 2% with PEG only. Additionally, plasmid encapsulation in Lipofectamine resulted in the highest cell viability percentage (45%) compared with PEG. Nine edited plants were obtained and identified based on the T7EI assay and Sanger sequencing. The developed edited lines exhibited downregulation of *CsNPR3* expression and upregulation of *CsPR1*.

**Conclusions:**

Our results demonstrate that utilization of the cationic lipid-based transfection agent Lipofectamine is a viable option for the successful delivery of donor DNA and subsequent successful genome editing in citrus.

**Supplementary Information:**

The online version contains supplementary material available at 10.1186/s13007-022-00870-6.

## Background

Citrus is an important fruit crop grown globally with many health benefits. However, it is also among the most difficult plants to improve through traditional breeding approaches due to its complex reproductive biology [1]. Because they lack pathogen resistance, commercial sweet orange cultivars are vulnerable to a plethora of diseases, including citrus canker resulting from *Xanthomonas axonopodis* infection and Huanglongbing (HLB) from the suspected causal agent *Candidatus Liberibacter asiaticus* (*Ca*Las), [2]. Genetic improvement of citrus using pathogen resistance genes that enhance plant defense mechanisms utilizing systemic acquired resistance (SAR) has resulted in the production of transgenic canker-resistant trees [3]. SAR employs the plant’s inherent pathogen defense system expressing specific defense genes to attenuate disease development and reduce pathogen spread [4]. The most well-studied regulator of SAR, Nonexpressor of *PR1* (*NPR1*), is also one of the most heavily researched transcriptional coactivators in the SA-dependent signaling pathway [4, 5]. *NPR3* represses *NPR1* function by enhancing *NPR1* degradation and decreasing defense pathway activation [6].

Genome editing by CRISPR/Cas9 is an emerging technology in plant improvement genetics. Using the Cas9 protein, we can precisely modify a targeted location by deletion, replacement, or insertion to develop novel traits. The application of genome editing requires synthetic nucleases that introduce double-strand breaks (DBSs) into a specified region of the genome and the natural DNA repair machinery that causes modifications [7]. Although there has been tremendous progress in plant development using CRISPR/Cas9, substantial efforts are still needed in this area to develop new methodologies that enhance transformation efficiency. CRISPR plasmid delivery into plant cells has also been limited to *Agrobacterium*-mediated delivery or particle bombardment [8]. However, challenges remain, such as the cell’s recalcitrance to gene editing, its inefficiency, or the difficulty of cell regeneration. CRISPR/Cas9-modified protoplasts are a viable option to modify citrus plants.

Protoplasts are often utilized for the development of genetically modified plants by the in vitro integration of recombinant DNA (either plasmid-based or linear) into the plant genome. The polyethylene glycol (PEG)-mediated method is widely used for protoplasts; however, it has a low transformation efficiency [9]. This low transformation efficiency occurs for many reasons, including the endosomal entrapment of donor DNA [10–12], degradation of donor DNA by cytosolic nucleases [13], or limitation of nuclear translocation due to the charge or size of the donor DNA [14, 15]. Other factors affecting the transformation of citrus protoplasts include the sensitivity of protoplasts to osmotic stress, PEG cytotoxicity, the type of plasmid construct, the physiological conditions of the cultivar, the low yield of protoplasts, and enzymatic degradation of the DNA construct prior to nuclear translocation due to prolonged exposure of plasmid DNA to the cytoplasm [16].

Novel methods to overcome cellular challenges can increase protoplast transformation efficiency and result in more viable cells with integrated donor DNA in their genomes. Donor DNA, siRNA, and proteins have been successfully delivered into animal and plant cells using viral vectors, lipofections and nanoparticles [17–21]. Lipofection (or liposome transfection) is a technique for delivering genetic material into a cell via liposomes, which can easily merge with the cell membrane since they are both made of a phospholipid bilayer. Nanostructured lipid carriers (NLCs), with a size ≤ 100 nm, are a potential delivery system that may have some advantages in certain circumstances when compared with other colloidal carriers [22]. Nanostructured lipid carriers have been utilized to encapsulate and deliver DNA to protect it from cellular nucleases, thus enhancing translocation of the DNA into the nucleus [23, 24]. The use of these chemicals depends on their ability to form lipoplexes with negatively charged donor DNA molecules [25, 26]. Lipofection uses cationic lipids to completely neutralize the charge of the CRISPR reagent. This results in a net positive charge that can relatively easily associate with the negatively charged surface of cells, resulting in uptake and intracellular distribution of the complex [27, 28]. However, this approach still needs optimization and is likely plant- and tissue type-dependent.

In addition to nanoparticles, cell penetrating peptides (CPPs) can also aid in the delivery of donor DNA into cells [29, 30]. CPPs are short peptides of no more than 30 hydrophobic amino acids [31]. Arginine-rich peptides and amphipathic peptides are the two most widely used peptides that aid in efficient DNA contract translocation into the nucleus [32–34]. These arginine-rich peptides have also been used in plant systems for protein delivery into onion (*Allium cepa* L.) and tomato (*Solanum lycopersicum* L.) [35] and for delivery of plasmid DNA into the nucleus of intact mung bean roots (*Vigna radiata* L.) and soybean (*Glycine max* L.) [36]. CPPs can be used on their own or in conjunction with polymer-based or lipid-based nanoparticles [37, 38]. CRISPR/Cas9 editing technology has been successfully utilized in citrus cells [39]. However, genome editing of citrus protoplasts is challenging, and stable genome-edited citrus using protoplasts has not been previously produced. Previous methods using PEG have proven to be cytotoxic to protoplasts [40]; therefore, new methods of transfection are needed to improve transfection efficiency without sacrificing cell health.

In this study we outline a new protocol for protoplast transfection using Lipofectamine™ LTX Reagent with PLUS Reagent to ensure donor DNA delivery in sweet orange protoplasts with low cytotoxic effects on the cells. We utilized this newly developed protocol to successfully edit the *CsNPR3* gene using a CRISPR/Cas9 construct and regenerate stable genome edited sweet orange citrus plants.

## Methods

### Cell culture and protoplast isolation

**Fig. 1:**
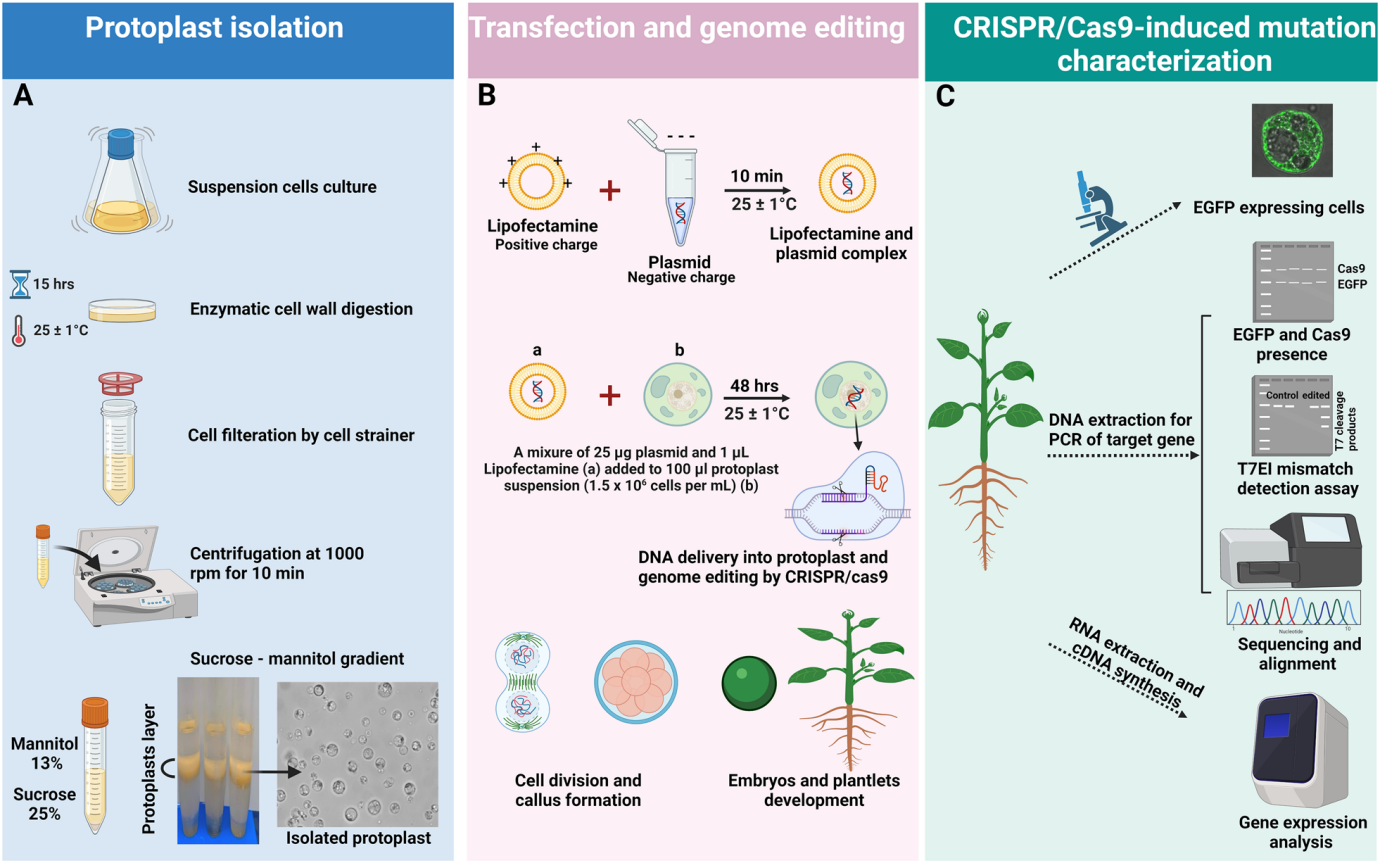
Schematic illustration of protoplast isolation and CRISPR/Cas9-mediated genome-editing steps via Lipofectamine. Protoplasts isolated from in vitro grown cell suspension (**A**). Protoplast layer represents protoplasts between solutions of sucrose 25% and mannitol 13%. Bright field of protoplast 1 h after isolation. Mixing of lipofectamine and plasmid constructs targeting *NPR3* and protoplast transfection (**B**). Characterization steps by monitoring EGFP expression and cas9 presence in the genomic DNA, T7EI assay and sanger sequencing and gene expression analysis (**C**). GFP-expressed cell was monitored by a confocal laser scanning microscope. The figure was created in BioRender.com

All the experiments in this study were conducted using the sweet orange (*Citrus sinensis* L. Osbeck) cultivar ‘N7-3’. ‘N7-3’ is a seedless sweet orange, which restricts its use in other forms of juvenile transformation systems [41]. Embryogenic calli and suspension cultures were established and maintained as previously described [42, 43]. Suspended cells were subcultured at 14 day intervals in modified H + H medium [44]. Cells were digested in protoplast incubation medium containing 0.6 M mannitol, 10 mM CaCl_2_, 10 mM MES buffer, 0.75% (w/v) Cellulase Onozuka RS (Yakult Honsha, Tokyo, Japan), 0.75% (w/v) Macerozyme R-10 (Yakult Honsha, Tokyo, Japan), pH 5.6, at 25 ± 1 °C for 15 h. The digested cells were filtered by a sterile cell strainer with 100 µM nylon mesh (Thermo Fisher Scientific, Waltham, MA). Protoplasts were collected by centrifugation on a sucrose-mannitol gradient and maintained as outlined in Fig. 1. The protoplasts were counted with a hemocytometer and then diluted to the desired concentration with BH3 media [42].

### Plasmid construction

The *CsNPR3* DNA sequence (orange 1.1g007849m) was identified from the *Citrus sinensis* genome assembly maintained in Phytozome (https://phytozome-next.jgi.doe.gov) by blasting *AtNPR3* (AT5G45110) against the genome. A gRNA specific to *CsNPR3* (TGATGAGAACACTGCAGTTGAGG) that was predicted to have low off-target effects was obtained using the online tool found at CRISPR-direct (http://crispr.dbcls.jp/ [45]). The chemically synthesized gRNA was placed under the control of the *Arabidopsis* U6–26 promoter in a transformation construct based on the pCAMBIA2300-EGFP vector [46] and containing the 35S—Cas9 transgene that had been codon optimized for *Arabidopsis* and contained two nuclear localization sequences—one at the 5ʹ end and the other at the 3ʹ end. Sanger sequencing was used to verify the resulting binary vector construct (pEN-NPR3) (Additional file 1: Fig. S1). NEB 10-beta competent *E. coli* (New England Biolabs, Ipswich, MA) was transformed with the binary vector and plated overnight to allow the bacteria to grow. Pure plasmid DNA was extracted using a Qiagen EndoFree Plasmid Maxi Kit (QIAGEN Sciences Inc., Germantown, MD) for all subsequent experiments.

### Evaluation of cationic lipid transfection and cell-penetrating peptides (C3POs)

Cationic lipid transfection reagents were evaluated for their efficacy in protoplast transformation by observing enhanced green fluorescent protein (EGFP) expression and cell morphology. Protoplasts (1.5 × 10^6^ cells per mL) were resuspended in a 1:1 (v:v) mixture of 0.6 M BH3 and 0.6 M EME sucrose in a Thermo Scientific™ BioLite 6-well plate (Thermo Fisher Scientific, Waltham, MA).

Several commercially available DNA transfection reagents were tested for DNA delivery into citrus protoplasts, including Chariot™ Protein Delivery Reagent (Active motif, Carlsbad, California), Escort™ (Sigma–Aldrich, St. Louis, MO), FuGENE® (Promega, Madison, Wisconsin, USA), Lipofectamine® 3000 (Thermo Fisher Scientific, Waltham, MA), Lipofectamine™ LTX with PLUS (Thermo Fisher Scientific, Waltham, MA), TransIT®-2020 (Mirus, Madison, WI), and Xfect™ (Takara Bio USA Inc., Mountain View, CA). All transfection reagents were initially evaluated according to the manufacturer’s standard protocol to determine the best cationic lipid for citrus protoplast transformation (Table 1). Lipofectamine LTX with PLUS reagent was subsequently chosen for the remainder of the study.

**Table 1:** Different transfection reagents were assessed for transfection efficiency in protoplasts indicated by EGFP presence

Transfection reagent	Concentration^a^	EGFP presence	Cell health
Chariot™	0.25 µl	Yes	+ ^b^
Escort™	25 µl	No	+
FuGENE®	5.0 µl	No	+ +
Lipofectamine® 3000	0.5 µl	Yes	+ +
Lipofectamine® LTX with plus™ reagent	0.5 µl	Yes	+ + +
TransIT®-2020	1.0 µl	No	+ +
X-fect™	7.5 µl	No	+

^a^Represents the concentration of transfection reagents mixed with 25 µg of plasmid DNA/ 100 protoplast (1.5 × 106 cells per mL)

^b^The effects of the transfection reagents on protoplast cell health and division were assessed. Poor (less than 20% intact protoplasts; +), good (over 50% intact protoplasts; + +), excellent (over 90% intact protoplasts; +  + +)

Small cell-penetrating peptides (CPPs) have been shown to aid in transfection. Arginine^9^ conjugated with TAMARA (Arg^9^-TAMRA) was used to evaluate whether CPPs improved the transformation efficiency. A 1 mg/ml stock solution was made by dissolving Arg^9^-TAMRA (Anaspec, Fremont, CA) in deionized water and stored at − 20 °C. In a preliminary study, Lipofectamine was tested with or without Arg^9^-TAMRA. Lipofectamine LTX and PLUS (0.5%) were mixed with 25 µg of plasmid DNA. The mixture was incubated for 10 min at room temperature to allow for DNA-lipid complex (lipoplex) formation. A total of 90 μl of Arg^9^ was added to each well. Cells were maintained in the dark at 25 ± 1 °C for 24 h before evaluation.

### Polyethylene glycol and lipofectamine-mediated CRISPR/Cas9 plasmid delivery

CRISPR/Cas9 plasmid delivery was tested with the use of Lipofectamine LTX Reagent with PLUS reagent (Thermo Fisher Scientific, Waltham, MA) alone or combined with polyethylene glycol (PEG). Lipofectamine LTX and PLUS (0.5%, 1%, 2%, or 4%) were mixed briefly with 25, 50, 75 and 100 µg of plasmid DNA, added to 100 µl of protoplasts at 1.5 × 10^6^ cells per mL, and incubated for 10 min. When Lipofectamine was applied alone, the diluted transfection reagents were added to the tube containing the CRISPR/Cas9 plasmid, followed by incubation at room temperature for 10 min. Then, the mixture was applied to 100 µl of protoplasts, mixed gently and incubated for 48 h. After 48 h, the transfection reagents and plasmid were removed by centrifugation at 1000 rpm for 2 min. For PEG treatments, 100 µl of protoplasts was added to the transfection mixture and incubated at room temperature for 15 min. A total of 100 µl of freshly prepared PEG solution (60% (W/V) PEG 6000, 0.4 M mannitol, 0.1 M CaCl_2_) was added to the protoplasts in a 2.0 ml Eppendorf tube and incubated at room temperature for 25 min in the dark. After incubation, the protoplasts were washed twice with 500 µl of BH3 liquid media. The protoplasts were pelleted by centrifugation at 1000 rpm for 5 min. The protoplasts were gently resuspended in 700 µl of 0.6 M BH3 and 0.6 M EME sucrose at a 1:1 ratio in a small 60 × 15 mm Petri dish, and the dishes were kept in the dark at 25 ± 1 °C for 48 h before EGFP was observed to evaluate the transfection efficiency. Each experiment was repeated six times.

### Protoplast regeneration and embryo initialization

Transfected protoplasts were resuspended in liquid media containing 0.6 M BH3:EME maltose at a 1:2 ratio and cultured for 30 days. The protoplasts were embedded in EME maltose semisolid medium containing 50 g/l maltose and 0.5 g/l malt extract and cultured in 25 ml petri dishes at 25 ± 1 °C in the dark. Subsequently, the cells were fed a few drops of the same media mixture (1:2) at three-week intervals. After 4–8 weeks, the cultures were transferred to the light (16 h light [30 μmol/m^2^/s] and 8 h darkness) and cultured at 25 ± 1 °C until microcallus and globular embryo formation. Induction of globular embryos was usually observed after approximately 1–2 months on EME-maltose medium.

Globular embryos visually observed to express EGFP were transferred to fresh semisolid EME-maltose medium and cultured over 0.2 µM sterilized cellulose acetate membrane filters to allow for embryo growth. Small torpedo stage transgenic embryos were subsequently enlarged on EME-1500 semisolid medium supplemented with 50 g/l sucrose and 1.5 g/l malt extract. For further embryo maturation and germination, the embryos were moved to B + medium containing 25 g/l sucrose, 0.10 µM NAA and 2.89 µM GA3. Shoots that formed were transferred for further root development and growth into RMAN rooting medium supplemented with 0.10 µM NAA. All plant growth regulator stocks were purchased from PhytoTech Labs, Lenexa, KS. Plantlets were subsequently micro grafted onto UFR15 rootstock [47] according to Dutt and Grosser [46]. Plants were acclimated following transfer to the greenhouse for further morphological, biochemical, and molecular analyses. All media formulations were prepared as previously outlined [42].

### Protoplast viability evaluation and transformation efficiency

Protoplasts were observed for cell morphology and putative pEN-*NPR3* plasmid transformation 48 h after transfection as indicated by EGFP expression under brightfield microscopy and EGFP fluorescence using a Zeiss Scope A1 fluorescence microscope (Carl Zeiss Microscopy, Gottingen, Germany) equipped with a FITC filter. Cell health was reported based on morphological observations in response to transfection reagents and was expressed qualitatively using the following symbols: + (poor; less than 20% intact protoplasts), +  + (good, over 50% intact protoplasts) and +  +  + (excellent; over 90% intact protoplasts). A Leica SP8 confocal microscope (Leica Microsystems, Wetzlar, Germany) was used to visualize the penetration and localization of the ARG^9^-TAMRA CPP. Protoplast nuclei were counterstained with DAPI (0.5 mg/ml) diluted 1:250 in protoplast media. DAPI was visualized using a 405-diode laser, and the TAMRA signal was visualized using a HeNe 561 laser. Images were captured with Leica’s LASX software (Leica Microsystems, Wetzlar, Germany).

Viability was monitored, and images were recorded 48 h after transfection. The number of viable cells was counted using a Countess II automated cell counter (Thermo Fisher Scientific, Waltham, MA) to determine the cell viability percentage [Cell viability % = (Number of viable cells/Total number of cells) × 100]. The transformation efficiency was calculated as the percentage of EGFP-expressing cells out of the total number of viable cells within 48 h of each treatment. [Transformation efficiency % = (Number of EGFP-expressing cells/Total number of viable cells) × 100].

### Detection of CRISPR/Cas9-induced mutations

Genomic DNA was isolated from the regenerated plants in three biological replicates using the GeneJET Plant Genomic DNA Purification Mini Kit (Thermo Fisher Scientific, Waltham, MA) following the manufacturer’s protocol. To confirm the presence of the Cas9 and EGFP transgenes in the putative edited embryos, duplex PCR was carried out in a thermal cycler (C1000; Bio–Rad Laboratories, Hercules, CA) using GoTaq Green Master PCR Mix (Promega Corp, Madison WI) and primers that amplified the Cas9 and EGFP genes.

Genomic DNA was used as a template for T7 endonuclease I (T7EI) mismatch detection assays. Primers were designed to amplify around the target region of *CsNPR3* using 10 ng of plant genomic DNA by Q5 High-Fidelity DNA polymerase (Thermo Fisher Scientific, Waltham, MA). The PCR products were purified using a PureLink Quick PCR Purification Kit (Invitrogen, Thermo Fisher Scientific) and quantified using a NanoDrop 2000c spectrophotometer (Thermo Fisher Scientific, Waltham, MA). A T7EI assay was used to evaluate 1 μg of the purified PCR product. The T7EI assay was performed according to the manufacturer’s instructions (New England Biolabs, Ipswich, MA). Briefly, the PCR products were denatured at 95 °C, and reannealing was carried out by ramp PCR from 95 to 85 °C at − 2 °C/s and 85 to 25 °C at − 0.1 °C/s. These annealed PCR products were incubated with T7 endonuclease I (New England Biolabs, Ipswich, MA) at 37 °C for 15 min and analyzed via 2% (w/v) agarose gel electrophoresis. PCR products were cloned into the pJET plasmid vector using a CloneJET PCR Cloning Kit (Thermo Fisher Scientific, Waltham, MA) and sequenced by the Sanger method. Ten colonies for each sample were used for colony sequencing. The sequencing results were compared with the sequence of the *CsNPR3* gene by alignment using the SnapGene program version 5.3.2 (SnapGene, San Diego, CA). All primer sequences are outlined in Additional file 1: Table S1.

### Gene expression assessment

Total RNA was isolated from 100 mg of leaf tissues using the Direct-zol™ RNA Miniprep Plus Kit (Zymo Research, Tustin, CA) according to the manufacturer’s protocol. cDNA was synthesized at 42 °C for 60 min by the RevertAid First Strand cDNA Synthesis Kit (Thermo Fisher Scientific, Waltham, MA) and quantified by a Nanodrop 2000c spectrophotometer. For RT–qPCR, 40 ng cDNA was added to PowerUp SYBR Green Master Mix for a final volume of 20 μl (Applied Biosystems, Foster City, CA) according to the manufacturer’s instructions. Each sample was tested twice in three replicates, and the data were analyzed using Applied Biosystems software version 3.0.1 (Thermo Fisher Scientific, Applied Biosystems, Foster City, CA). The Ct value of the PCR curve was analyzed and compared with that of the wild type. The relative expression of the selected gene was calculated by the 2^−ΔΔCT^ method [48]. Actin was used as a housekeeping gene to provide an endogenous control. Transcript levels of the *CsNPR3* and *CsPR1* genes were evaluated.

### Statistical analysis

Analysis of variance (ANOVA) was conducted in JMP Pro version 15 (SAS Institute, Cary, NC, USA). The combinations of Lipofectamine and DNA concentrations were designed as a factorial experiment with two factors (Lipofectamine concentrations (four levels) and DNA concentrations (four levels)). The effect of transfection agents on the number of EGFP-positive cells and cell viability and the relative expression data were analyzed using one-way ANOVA. Each experiment was repeated six times with three replicates, and each replicate represented four plates. Tukey’s method was used to compare means among the samples. Differences were significant when *p* values were less than 0.05%.

## Results

### Lipoplex-mediated DNA delivery into citrus protoplasts

**Fig. 2:**
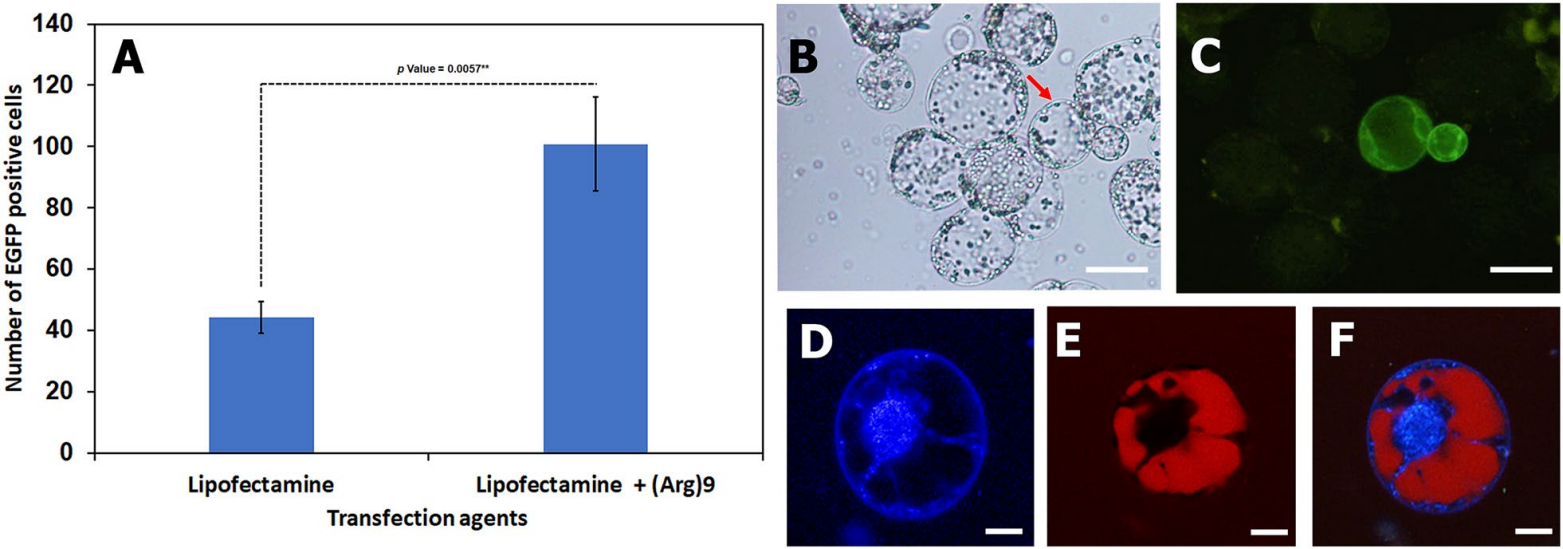
Transient gene expression in *Citrus sinensis* protoplasts. The average number of EGFP positive cells in ‘N7-3’ protoplast cultures were greater in those treated with Lipofectamine LTX + the Arg9 CCP compared to those treated with Lipofectamine LTX only (**A**). Error bars represent standard error. Brightfield (**B**) and fluorescent (**C**) images showed that some of the protoplasts were EGFP positive 72 h after transfection. Confocal image of a protoplast cell indicating that the Arg9 CPP conjugated with TAMRA penetrated the cell membrane but not the nuclear membrane. There was no colocalization between the nucleus stained with DAPI (**D**) and the TAMARA signal (**E**) indicated by the merge (**F**). Scale bar indicates 100 µM in length

Several transfection reagents were evaluated for their transformation efficiency and their effects on cell health at different concentrations (Table 1). Based on these results, only 0.25 µl Chariot, 0.5 µl Lipofectamine 3000 and 0.5 µl Lipofectamine LTX mixed with 25 µg of plasmid DNA/100 protoplast (1.5 × 106 cells per mL) produced EGFP-expressing cells. Lipofectamine LTX outperformed the other transfection reagents, resulting in a high transformation efficiency without compromising cell health and producing over 90% intact protoplasts (Fig. 2). Cell-penetrating peptides have been shown to aid in donor DNA delivery into the cell; therefore, the transfection efficiency of Lipofectamine LTX was compared with and without the Arg^9^ CPP. Lipofectamine LTX coupled to CPP Arg^9^ resulted in more EGFP-positive cells, indicating a higher transfection efficiency than Lipofectamine LTX alone (Fig. 2A).

While lipoplexes conjugated to Arg^9^ CPP increase the transfection efficiency, less is known about their cellular mechanisms. Accordingly, we examined the behavior of lipoplexes and CPPs inside the cell. The CPP did not enter the nucleus when Arg^9^ CPP conjugated to a TAMRA reporter was transfected into citrus protoplasts (Fig. 2D–F). Additionally, plants were not generated from these transfection attempts, as the cells failed to divide and produce microcalli.

### Optimization of lipofectamine-mediated DNA delivery into citrus protoplasts

**Fig. 3:**
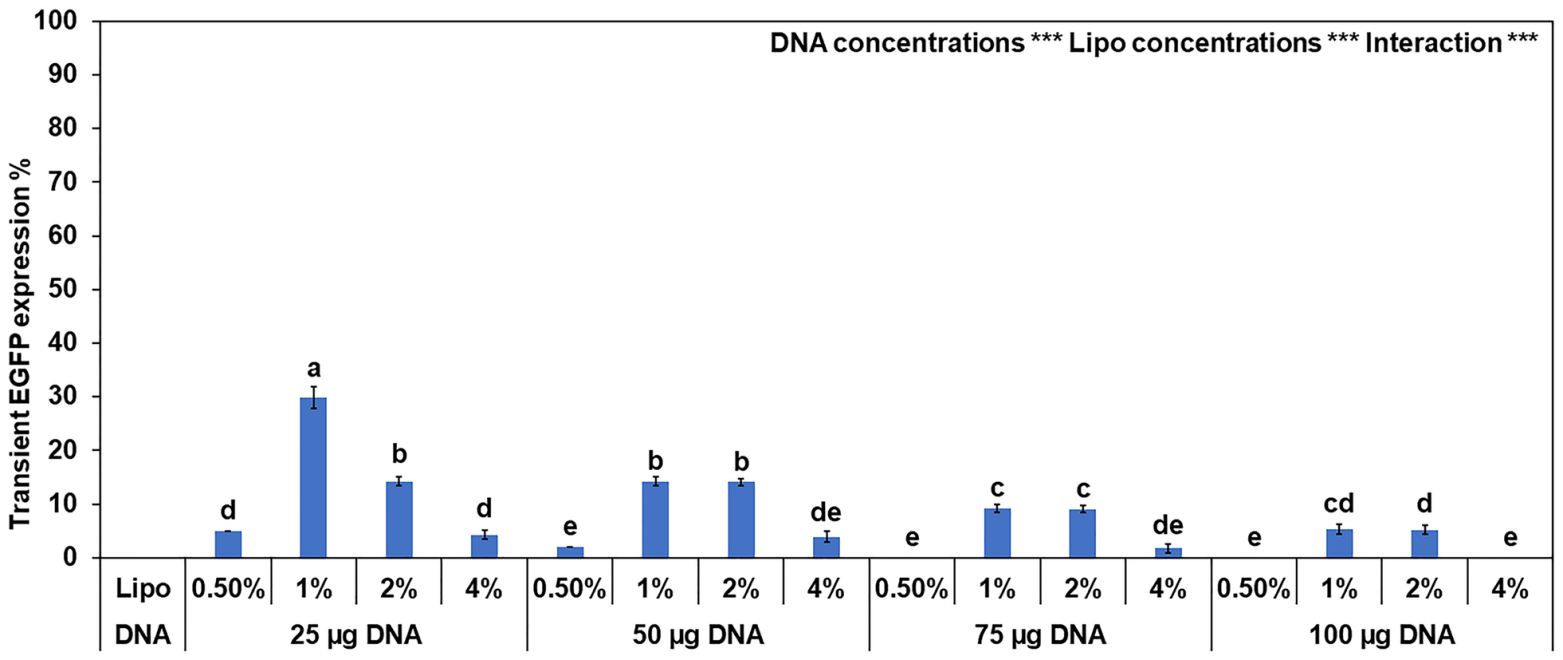
Effect of different concentrations of CRISPR/Cas9 plasmid encapsulated in different amounts of Lipofectamine (Lipo) mediated DNA delivery into citrus protoplasts. Four concentrations of DNA were used (25, 50, 75 and 100 µg) and four amounts of Lipo (0.5, 1, 2 and 4%). ***Expressed *p* value < 0.0001. Transfection efficiency was calculated as the percentage of GFP-expressed cells by the total number of the viable cells within 48 h of each treatment ± standard errors (vertical bars). Different letters represent significant differences by Tukey’s honestly test (*p* ≤ 0.05)

Lipofectamine LTX improved the transformation in our previous experiment and had few negative effects on cell health. Further optimization of the citrus protoplast transfection system was performed to target the *CsNPR3* gene and produce a population of genome-edited plants. The pEN-NPR3 construct was encapsulated in cationic lipid nanoparticles using Lipofectamine™ LTX Reagent supplemented with the manufacturer-provided PLUS Reagent, which has been reported to further enhance the transfection efficiency of Lipofectamine LTX [49]. We tested whether different DNA concentrations (25, 50, 75 and 100 µg) and different amounts of Lipofectamine LTX with PLUS (0.5, 1, 2, and 4%) would affect the transfection efficiency of CRISPR/Cas9 construct delivery into sweet orange protoplasts. It was observed that the lowest DNA concentration (25 µg) produced the best transformation efficiency (32%) when combined with 1% transfection reagent (Fig. 3).

**Fig. 4:**
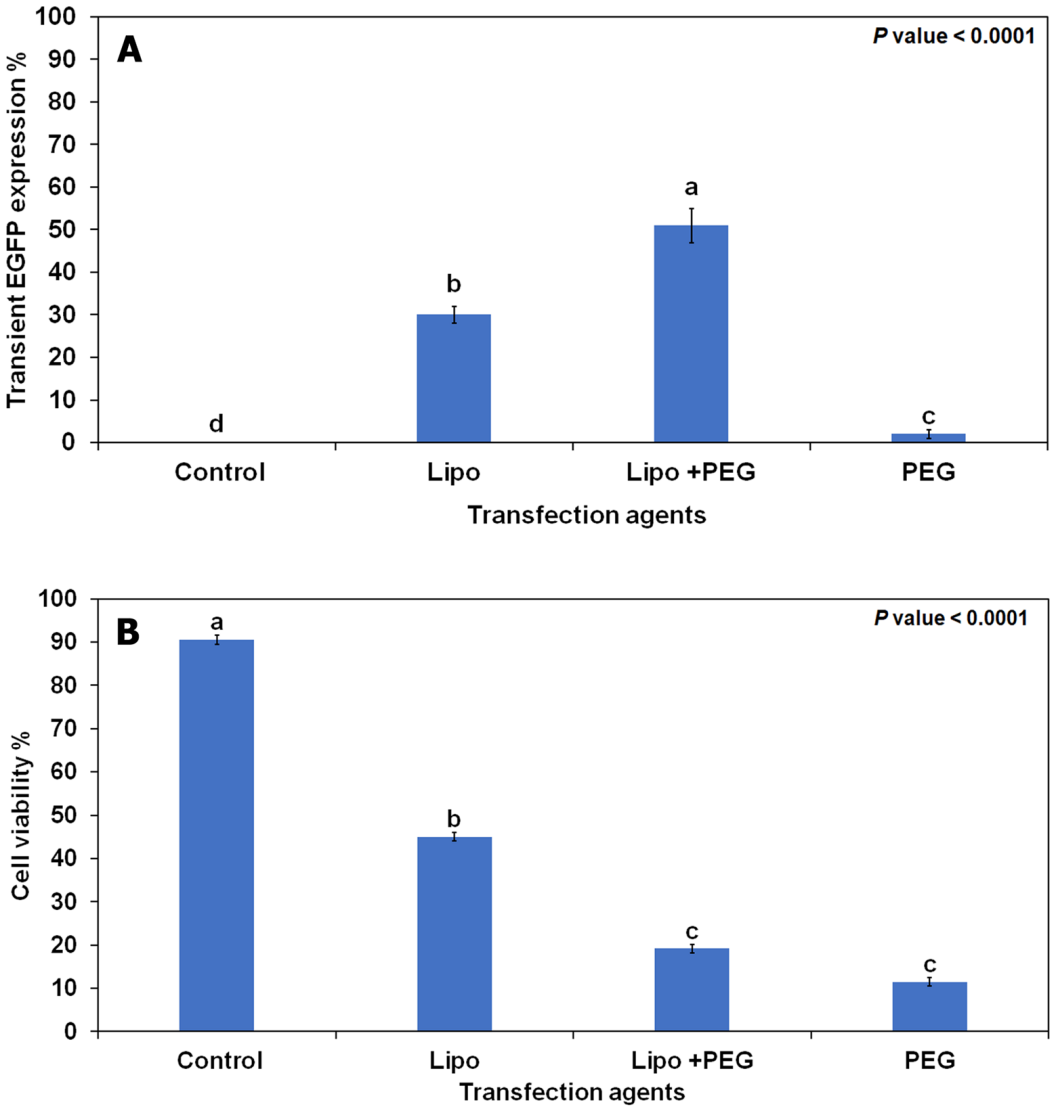
Evaluation of transfection agents on the transient transformation efficiency (**A**), and cell viability (**B**) of citrus protoplasts

**Fig. 5:**
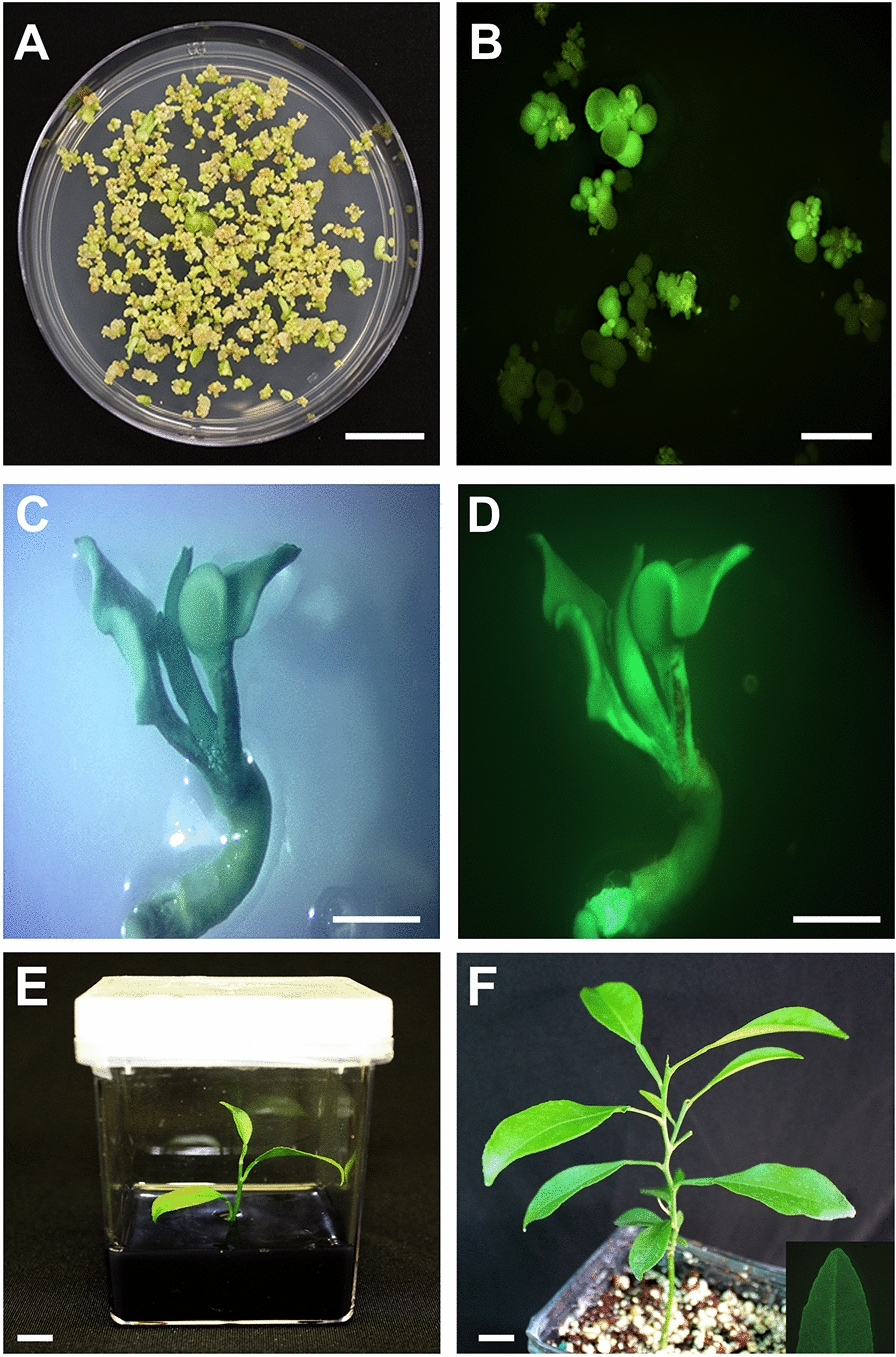
Development of genome edited transgenic plants following lipofectamine-mediated plasmid delivery into protoplast. **A** Colonies of developed embryos, (**B**) EGFP-expressing embryos. A germinating EGFP expressing seedling visualized under white light (**C**) and the same seedling exhibiting EGFP expression under an epi-fluorescence stereomicroscope (**D**). **E** Regenerated plants in tissue culture medium before micrografting and (**F**) Micrografted plant well acclimated in the greenhouse. Inset shows a section of the leaf exhibiting EGFP fluorescence as visualized under the dissecting microscope fitted with a NIGHTSEA fluorescence adapter. Scale bar indicates 1 cm in length

Protoplast transfection methods using PEG usually result in a low transfection efficiency, primarily due to PEG cytotoxicity. Accordingly, we tested whether using Lipofectamine in addition to polyethylene glycol (PEG) or PEG alone would affect DNA delivery and cell viability in citrus protoplasts. The transfection efficiency was approximately 30% using Lipofectamine alone and 51% when Lipofectamine was combined with PEG (Fig. 4A). Although using Lipofectamine combined with PEG increased transfection efficiency, low levels of cell viability were recorded when PEG was used alone or combined with Lipofectamine (11.50 and 19.16%, respectively) (Fig. 4B). Additionally, severe damage was observed in the transfected protoplasts using PEG (Additional file 1: Figure S2). In this study, we generated approximately 100 EGFP + embryos using our optimized protocol. After 6 weeks in B + germination medium, the germinating plants with a well-developed apical meristem were transferred to rooting medium (RMAN) for root production and growth (Fig. 5).

### Verification of gene editing in plant tissues using T7EI and sanger sequencing

**Fig. 6:**
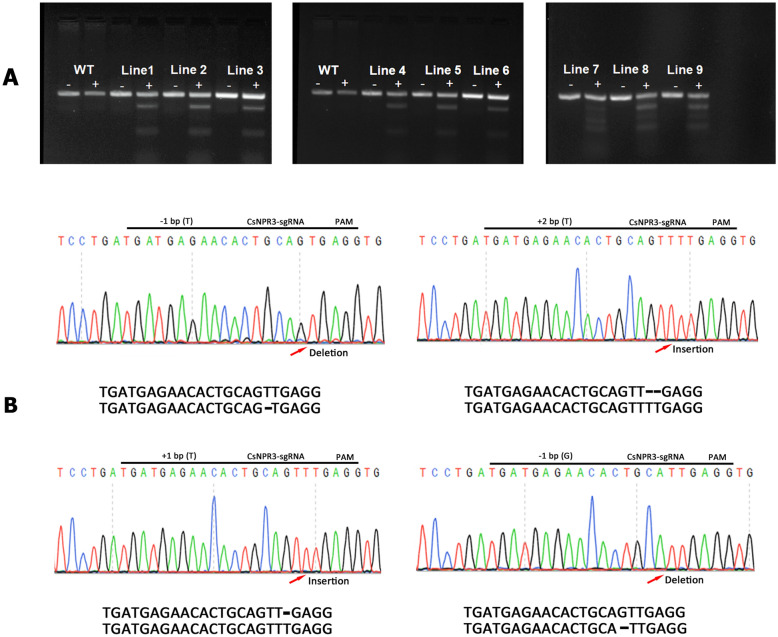
Targeted mutagenesis of *NPR3*. T7 Endonuclease I (T7EI) assay targeting *CsNPR3* indicating cleavage products from targeted mutagenesis (**A**). Positive sign ( +) is used to indicate T7EI reaction by incubate the annealed PCR product at 37 ºC for 15 min. The untreated samples are marked with negative sign (−). Sanger sequencing chromatograms of four selected lines. Arrows point to the editing site and the modifications are indicated alongside (**B**)

Following transfection and successful regeneration, genome editing was explored in citrus tissue. Twelve EGFP-positive plantlets were randomly selected for analysis, gDNA was isolated, and the presence of both the Cas9 and EGFP genes was tested. PCR analysis of the genomic DNA determined that nine lines tested positive for the presence of both the Cas9 and EGFP genes (Additional file 1: Figure S3). Mutations from 9 independent transgenic lines containing *NPR3*-sgRNA were also detected using the T7 endonuclease I (T7EI) assay (Fig. 6A). In the T7EI assay, DNA fragments with mutations were digested by the T7EI enzyme, whereas DNA fragments were not digested in the wild-type and untreated controls. The results were further verified by Sanger sequencing (Fig. 6B). The Sanger sequencing results of the PCR-amplified *NPR3*-gRNA sequence revealed that all nine transgenic plants carried at least one mutation in the *CsNPR3* gene. We commonly observed single or double base changes, such as addition of a T or deletions, such as T or G deletions, in the *CsNPR3* gene.

### Differential gene expression in the edited plants

**Fig. 7:**
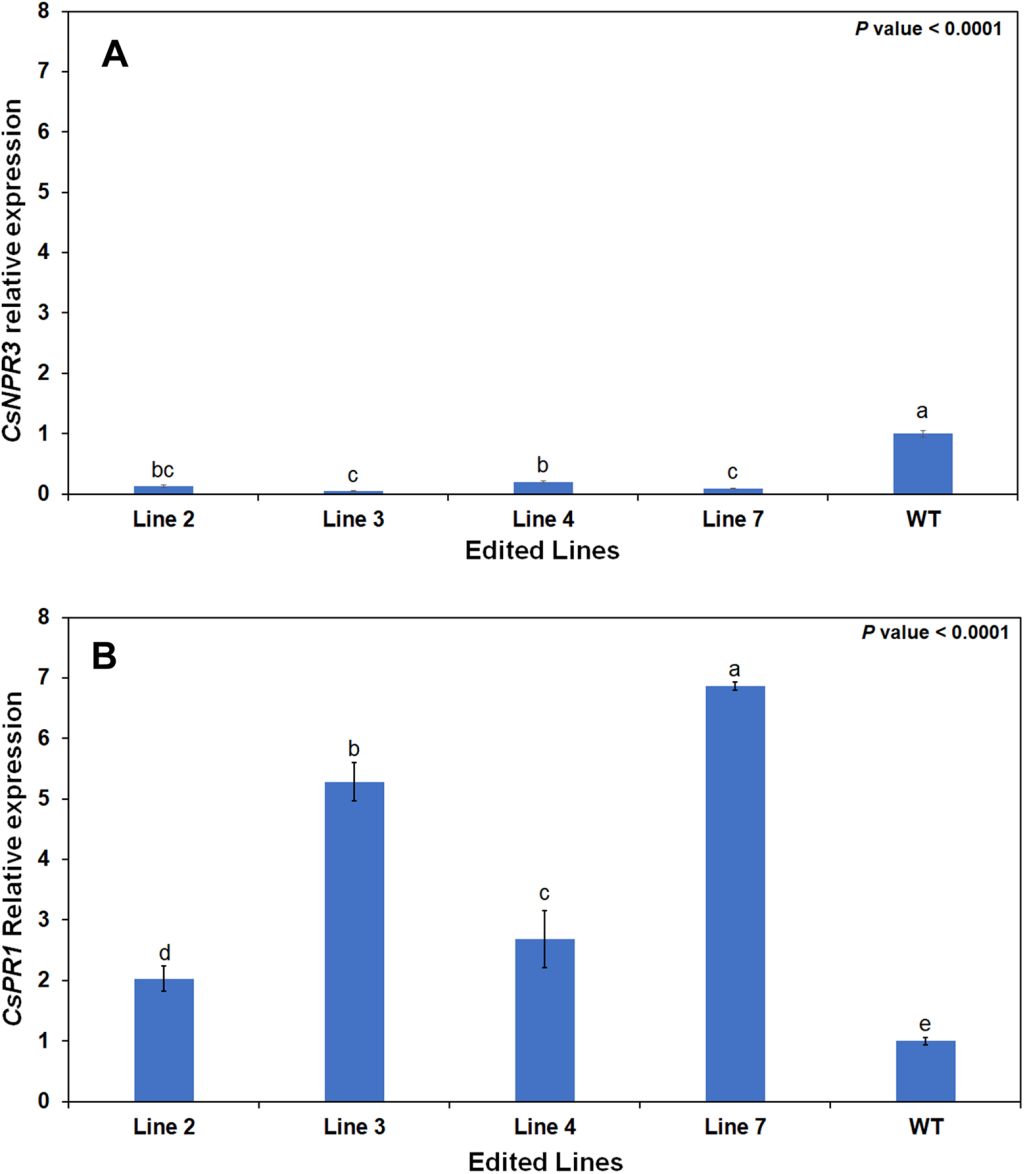
Changes in relative expression of *CsNPR3* (**A**), and *CsPR1* (**B**) of four selected and edited sweet orange ‘N7-3’ plants. Means compared using Tukey–Kramer HSD test, means followed by the same letter were not different at (*p* < 0.05)

The stable edited lines were micro grafted onto UFR15 [47] rootstocks for faster growth and subsequently transferred to the greenhouse for acclimatization. The transcript levels of *CsNPR3* and *CsPR1* were analyzed using RNA isolated from fully expanded leaf from four randomly selected edited plants. There was none to very little *CsNPR3* expression in all edited lines while *CsPR1* expression was significantly upregulated. Edited lines 3 and 7 had the highest expression, while the gene expression in lines 2 and 4 were lower but still statistically significant compared to control (Fig. 7)*.*

## Discussion

The CRISPR/Cas9 system has now been applied widely as a new approach to genome editing in many plant species, including citrus [50]. It can be challenging to compare the different transfection methods because of the many variables that control successful transfection and editing. The cell’s recalcitrance of gene editing and the difficulty of cell regeneration are the main factors that affect which approach is utilized. CRISPR plasmid delivery into citrus cells has been limited to *Agrobacterium*-mediated delivery [39, 50]. Most DNA insertion techniques rely on juvenile tissues, which can give rise to homozygous, heterozygous**,** biallelic, or chimeric edited plants [51]. Conversely, some factors could cause damage to the plasmid DNA, thus inducing cleavage under inappropriate environmental conditions or by DNAses [52]. In plant cells, the cell wall restricts the direct entry of DNA into the cytoplasm. However, protoplasts with their cell wall removed enzymatically are more amenable to the entry of DNA [53]. Since protoplasts give rise to plants derived from a single cell, there is a high possibility of producing homozygous edited plants, which are desirable in vegetatively propagated cultivars. However, due to the difficulty of achieving protoplast transfection in woody fruit trees, which are mainly limited by the cytotoxicity of PEG used in this process, there have been very few successful studies reporting the integration of donor DNA into woody tree protoplast genomes using CRISPR/Cas9 [54]. In citrus, even when using highly viable protoplasts from young embryogenic cultures, the transformation rate and production of stable transgenic plants remain low [55, 56]. If the cells are also negatively affected due to the cytotoxicity of the transfection reagent, then there will be fewer recombination events, thus lowering the transformation efficiency. We suggest herein a simplified method for the insertion of foreign DNA into the citrus cell and stable integration of the DNA into the citrus genome.

Accordingly, a protocol was developed to deliver DNA using cationic lipid nanoparticles into citrus protoplasts for successful gene editing. Polymeric nanoparticles and cationic lipid formulations work to encapsulate DNA, allowing them to escape cellular endosomal barriers and protecting them from enzymatic degradation [57–60]. Comparative studies have been conducted to further evaluate the performance of Lipofectamine and have demonstrated that the activity of lipid formulations is enhanced when coupled with cell penetrating peptides [38, 61]. Lipofectamine LTX was best suited for protoplast transfection and resulted in good EGFP expression. Additionally, when used at an optimum concentration, it had a negligible cytotoxic effect on the cells.

Previous studies have demonstrated that the activity of lipid formulations is enhanced when combined with cell penetrating peptides (CPPs) [37, 38]. These CPPs are a short group of amino acids designed to deliver nucleic acids [31, 62] and lipoplexes (lipid + DNA complexes) into the cell [63, 64]. Treatment with Lipofectamine LTX coupled to Arg^9^ resulted in more EGFP-positive cells than treatment with Lipofectamine LTX alone. These data are consistent with previous reports that adding arginine-rich CPPs to lipoplexes enhanced the transformation efficiency [63, 65–67]. CPPs not only enhance the transformation efficiency but also improve the stability of lipoplexes [68] and promote translocation, which results in higher levels of gene expression [63].

To investigate the behavior of CPPs and lipoplexes in citrus protoplasts, an Arg^9^ CPP conjugated with TAMARA was used to track the movement of the CPP inside the cell. We found that the CPP did not cross the nuclear envelope but remained in the cytoplasm. It is not known whether the CPP was cleaved and dissociated in the cytoplasm or if the complex was intact. However, our data indicate that CPPs result in increased transfection. It is possible that the Arg^9^ CPP dissociated from the DNA complex in the cytoplasm and that the donor DNA crossed the nuclear envelope to be integrated into the citrus genome. Even though CPPs improve the transfection efficiency, they may only increase the probability of genetic recombination events simply because donor DNA plasmids can cross the cell membrane and enter the cell. However, the presence of the Arg^9^ CPP may affect cell division given that it was difficult to generate stably edited plants from these transfected cells. Thus, the PLUS reagent, which is also widely used in mammalian transfection studies [69], was used in conjunction with Lipofectamine LTX for the remainder of the study, as it has been reported by the manufacturer to further improve Lipofectamine LTX transfection.

Integration of CRISPR/Cas9 construct and expression was validated and several mutations targeting the *CsNPR3* gene was obtained (Fig. 6). Successful DNA delivery and genome editing depend on many factors, including the efficiency of DNA delivery, cell viability maintenance and cellular toxicity. Polymeric nanoparticles and cationic lipid formulations work by encapsulating DNA [70]. An optimized Lipofectamine LTX Reagent with PLUS reagent protocol allowed for the stable transfection and gene editing of citrus protoplasts, which generated several stably edited cell lines. Additionally, the use of a lower plasmid DNA concentration (25 µg) resulted in the highest transfection efficiency. Once nanoparticles are introduced into the cell, they allow DNA to escape endosomal barriers and protect it from enzymatic degradation [57, 71]. DNA release from the DNA-polymer complex is essential for translocation and subsequently highlights a drawback of polyplex-mediated DNA delivery [72]. Thomas and Klibanov [73] have also demonstrated that the proper compaction of DNA into the polymer matrix is a prerequisite for the high transfection efficiency of polymer-based nanoparticles. Interestingly, encapsulation of the plasmid DNA in the lower concentration of Lipofectamine used in this study led to higher transfection efficiency. This result is most likely due to a reduction in reagent cytotoxicity to the cells, allowing for more healthy cells to take up and integrate the donor DNA. In some cases, the low transformation efficiency is caused by the endosomal entrapment of donor DNA [10–12], degradation of donor DNA by cytosolic nucleases [13], or limitation of nuclear translocation due to the charge or size of the donor DNA [14, 15]. The introduction of DNA encapsulated in Lipofectamine enhances the transfection efficiency because of the positive charge and maintains cell viability. An increase in the transfection efficiency was recorded when Lipofectamine was used with the addition of PEG. However, the addition of PEG resulted in decreased cell viability and cellular damage. If cellular health is poor following transfection, the downstream establishment of rapidly proliferating embryogenic masses and the subsequent production of plants will be difficult. Pathirana et al. [74] illustrated the effect of PEG in inducing osmotic stress conditions that resulted in changes in cellular and nuclear morphology, DNA fragmentation, cell plasma membrane permeabilization, reactive oxygen species (ROS) overproduction and severe oxidative stress. Additionally, electroporation and DNA carriers activate cellular DNA damage signaling pathways. The low toxicity, simplicity, and high efficiency of using Lipofectamine make it a viable alternative method to deliver DNA for the stable transformation of plant protoplasts. Using the protocol developed in this study, frame shift mutations were obtained through the insertion of 1 or 2 nucleotides or the deletion of 1 nucleotide as previously observed in a callus transformation study [39]. The *CsPR1* gene was upregulated in all CRISPR-edited lines. PR1 is used as a marker for the SAR induction process and is naturally upregulated in response to stress [75]. It is indeed possible that enhanced basal upregulation of the *CsPR1* gene in our edited lines could result in a robust defense response when the edited lines are challenged by different plant pathogens [76].

## Conclusion

We have developed a genome editing system that can effectively improve the efficiency of citrus protoplast transfection and cell viability by using lipid-encapsulated DNA compared to naked plasmid DNA and traditional PEG-mediated transformation. Genomic analysis confirmed the presence of mutations in the *CsNPR3* gene, and DNA fragments with mutations were successfully digested by the T7EI enzyme. The edited citrus plants for the SAR-related gene *CsNPR3* had enhanced *CsPR1* gene expression. This developed protocol can be used in further applications using CRISPR/Cas9 genome editing to improve plant growth and increase tolerance to biotic and abiotic stress.

## Supplementary Information

**Additional file 1: Table S1.** Primer sequences used in this study.

**Additional file 2: Figure S1.** A diagrammatic representation of the DNA construct used in this study targeting the CsNPR3 gene.

**Additional file 3: Figure S2.** Protoplast damage following transfection using polyethylene glycol (PEG). Red Arrow indicates intact protoplasts.

**Additional file 4: Figure S3.** Amplification products obtained from duplex PCR of transgenic ‘N7-3’ genomic DNA with gene-specific oligonucleotide primers. A 800 bp fragment of the Cas9 gene was amplified along with a 490 bp fragment of the EGFP gene. M, 1 kb marker.

## Data Availability

The datasets used and/or analyzed during the current study are available from the corresponding author on reasonable request.

## Abbreviations

CPP: Cell penetrating peptides
CRISPR: Clustered regularly interspaced short palindromic repeats
EGFP: Enhanced green fluorescent protein
FITC: Fluorescein Isothiocyanate
gRNA: Guide RNA
MES: 2-(*N*-Morpholino) ethanesulfonic acid
PCR: Polymerase chain reaction
PEG: Polyethylene glycol
ROS: Reactive oxygen species
RNA: Ribonucleic acid
SAR: Systemic acquired resistance
