# Supplementary material for: A cationic lipid mediated CRISPR/Cas9 technique for the production of stable genome edited citrus plants
Source: Plant Methods. 2022 Mar 18;18:33. doi: 10.1186/s13007-022-00870-6 (PMC8932238; doi:10.1186/s13007-022-00870-6)
Supplement: Supplementary file 1 — Additional file 1: Table S1. Primer sequences used in this study. [file 13007_2022_870_MOESM1_ESM.pdf]

Additional file 1: Table S1: Primer sequences used in this study.

**Seq Name**	**Purpose**		**Primer sequences 5'→3'**
CsNPR3	qPCR	Forward	TCCTTGCTCAATGTGTTGATAGA
		Reverse	AAGACTTGAGTCGGAGCATTC
CsNPR1	qPCR	Forward	GTAGGCCGGCTGTTGATTT
		Reverse	GTCTAGGAGGTGCCTCTGATAA
CsPR1	qPCR	Forward	CAGGGTCTCCAAGCAACTATG
		Reverse	CCACCTCGCGTATTTCTCTAA
CsActin	qPCR	Forward	GCTGCCTGATGGCCAGATC
		Reverse	AGTTGTAGGTAGTCTCATGAA
Cas9	PCR	Forward	GAAGCAGCTCAAAGAGGACTAC
		Reverse	GCTCTTGGTCCACGTACATATC
EGFP	PCR	Forward	CAAGACCAGAGCTGAGGTTAAG
		Reverse	GGATGTTTCCGTCCTCTTTGA
CsNPR3	T7EI/Sequencing	Forward	TTGGCTGCCTTCCATTGTCAATTAA
		Reverse	GAGTGCATTGGCTTCGTCTAAGG
