# Supplementary material for: A cationic lipid mediated CRISPR/Cas9 technique for the production of stable genome edited citrus plants
Source: Plant Methods. 2022 Mar 18;18:33. doi: 10.1186/s13007-022-00870-6 (PMC8932238; doi:10.1186/s13007-022-00870-6)
Supplement: Supplementary file 2 — Additional file 2: Figure S1. A diagrammatic representation of the DNA construct used in this study targeting the CsNPR3 gene. [file 13007_2022_870_MOESM2_ESM.pdf]

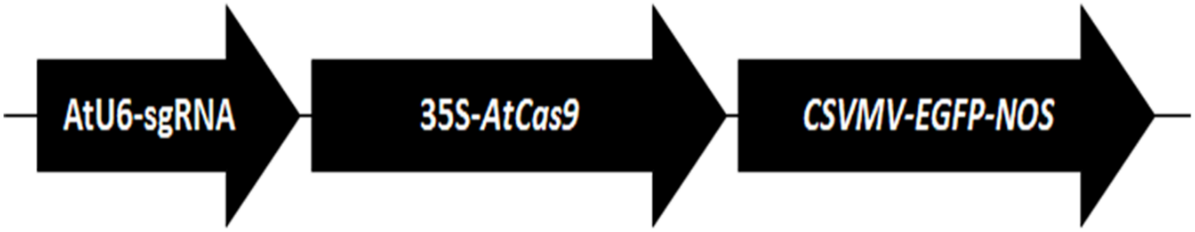

Additional file 2: Figure S1. A diagrammatic representation of the DNA construct used in this study targeting the CsNPR3 gene.
