# Supplementary material for: A cationic lipid mediated CRISPR/Cas9 technique for the production of stable genome edited citrus plants
Source: Plant Methods. 2022 Mar 18;18:33. doi: 10.1186/s13007-022-00870-6 (PMC8932238; doi:10.1186/s13007-022-00870-6)
Supplement: Supplementary file 3 — Additional file 3: Figure S2. Protoplast damage following transfection using polyethylene glycol (PEG). Red Arrow indicates intact protoplasts. [file 13007_2022_870_MOESM3_ESM.pdf]

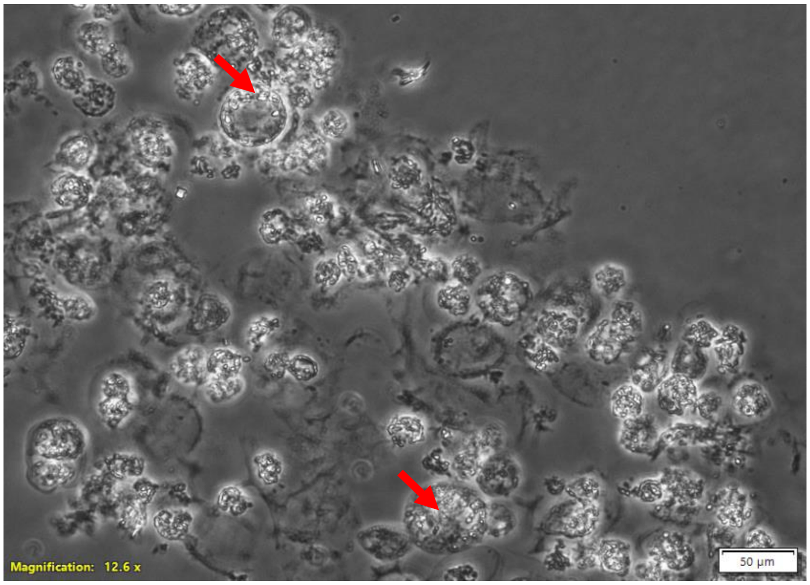

Additional file 3: Figure S2. Protoplast damage following transfection using polyethylene glycol (PEG). Red Arrow indicates intact protoplasts.
