# Supplementary material for: A cationic lipid mediated CRISPR/Cas9 technique for the production of stable genome edited citrus plants
Source: Plant Methods. 2022 Mar 18;18:33. doi: 10.1186/s13007-022-00870-6 (PMC8932238; doi:10.1186/s13007-022-00870-6)
Supplement: Supplementary file 4 — Additional file 4: Figure S3. Amplification products obtained from duplex PCR of transgenic ‘N7-3’ genomic DNA with gene-specific oligonucleotide primers. A 800 bp fragment of the Cas9 gene was amplified along with a 490 bp fragment of the EGFP gene. M, 1 kb marker. [file 13007_2022_870_MOESM4_ESM.pdf]

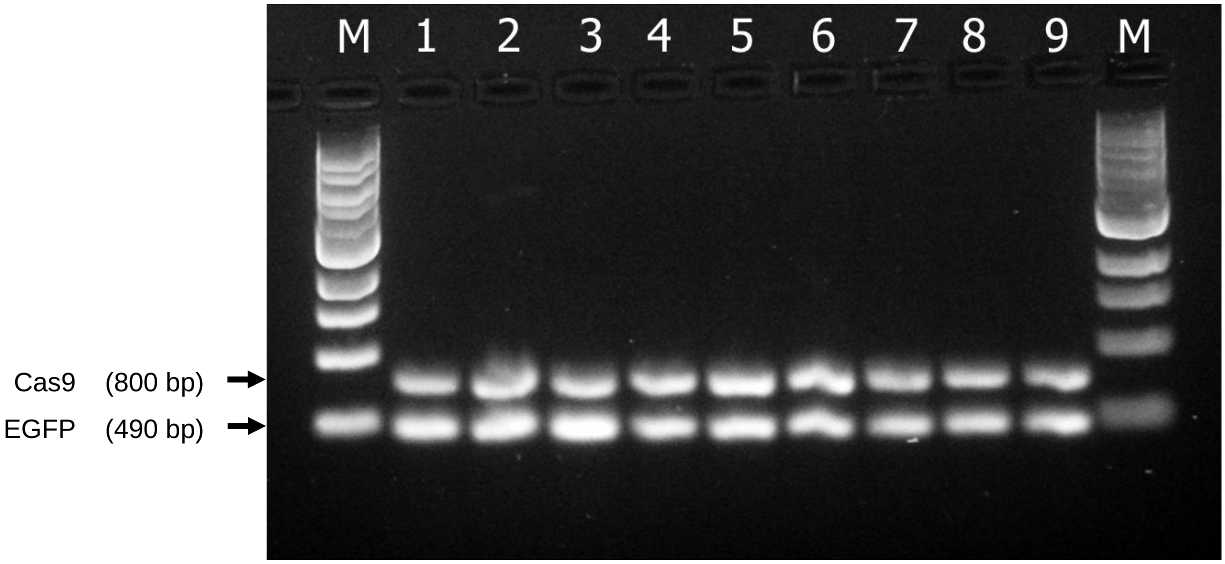

Additional file 4: Fig. S3. Amplification products obtained from duplex PCR of transgenic ‘N7-3’ genomic DNA with gene-specific oligonucleotide primers. A 800 bp fragment of the Cas9 gene was amplified along with a 490 bp fragment of the EGFP gene. M, 1 kb marker.
